# Cardiac Arrest as the First Presentation of Gitelman Syndrome

**DOI:** 10.7759/cureus.33565

**Published:** 2023-01-09

**Authors:** Abeselom Geletu, Jayna Gardner-Gray, Meaghan Roche, Marina Ngassa

**Affiliations:** 1 Internal Medicine, Henry Ford Health System, Detroit, USA; 2 Critical Care Medicine, Henry Ford Health System, Detroit, USA; 3 Nephrology, Henry Ford Health System, Detroit, USA

**Keywords:** metabolic alkalosis, bartter syndrome, implantable cardioverter-defibrillator (icd), cardiac arrythmia, familial hypokalemia-hypomagnesemia, gitelman syndrome

## Abstract

Gitelman syndrome is a salt-wasting tubulopathy characterized by profound hypokalemia, hypomagnesemia, metabolic alkalosis, and hypocalciuria. Cardiac arrest is a relatively rare manifestation of Gitelman syndrome. Here we present a case of Gitelman syndrome in a patient with recurrent cardiac arrest. A 43-year-old female was admitted for out-of-hospital cardiac arrest secondary to ventricular fibrillation. Initial workup revealed severe hypokalemia, hypomagnesemia, metabolic alkalosis, and prolonged QTc. The workup revealed a picture of salt-wasting tubulopathy with hypokalemia, hypomagnesemia, and hypocalciuria. Potassium was repleted aggressively, and the patient received potassium-sparing agents resulting in the stabilization of potassium levels. Before discharge, an implantable cardioverter defibrillator (ICD) was placed for secondary prevention of cardiac arrest. The patient remained symptom-free, and electrolytes remained stable. This case highlights the diagnostic challenges of Gitelman syndrome and the importance of accurate diagnosis in improving patient outcomes.

## Introduction

Gitelman syndrome (GS) was first described in the 1960s as a familial renal impairment in the conservation of potassium and magnesium, which leads to a state of hypokalemia, hypomagnesemia, hypocalciuria, and metabolic alkalosis [1]. Disease severity varies greatly even amongst family members and manifests with a constellation of non-specific symptoms such as fatigue, muscle weakness, muscle spasms, dizziness, and/or fainting. Hence, the disease is frequently misdiagnosed or unnoticed, making estimation of true prevalence difficult. The estimated prevalence in the white population is about one in 40,000. Although generally deemed a benign or asymptomatic condition [2], dangerous arrhythmias may occur [2,3], leading to sudden cardiac arrest. Given the non-specificity of presenting symptoms and spectrum of disease severity, it remains a diagnostic difficulty if not missed in diagnosis entirely. This report presents a case of GS presenting as recurrent ventricular fibrillation cardiac arrest.

## Case presentation

A 43-year-old female with a prior history of recent cardiac arrest, seizure disorder, and cerebral aneurysm, was admitted to the medical intensive care unit after an out-of-hospital cardiac arrest. She was in her usual state of health a few hours prior to her presentation when she was found by a family member unresponsive and seizing, then ultimately pulseless. She immediately received bystander cardiopulmonary resuscitation (CPR) until emergency medical services (EMS) arrived. On EMS arrival, ventricular fibrillation (VFib) was appreciated on an automated external defibrillator. She was defibrillated, and amiodarone was administered with a successful return of spontaneous circulation. On arrival to the emergency department, examination revealed a temperature of 36.9^o^C, irregular heart rate of 95 beats per minute, blood pressure of 83/59 mmHg, and respiratory rate of 22 breaths per minute, and she had a Glasgow Coma Scale score of 5, which lead to urgent intubation.

Collateral history revealed that the patient had a similar cardiac arrest six months ago when she had a seizure activity followed by VFib cardiac arrest. At that time, her admission workup was significant for hypokalemia and hypomagnesemia with prolonged QTc on an electrocardiogram.

**Figure 1:**
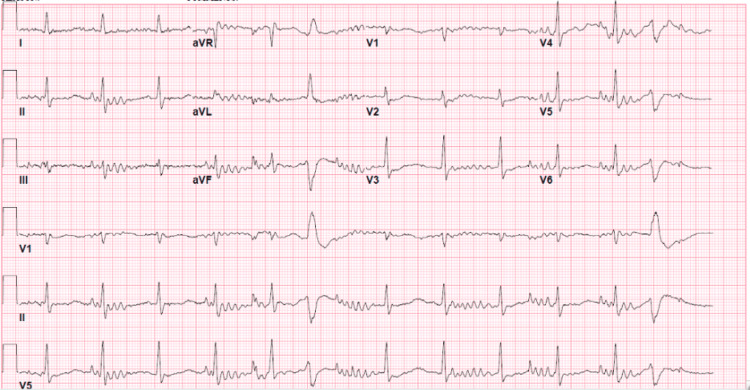
Electrocardiogram showing undetermined rhythm, nonspecific ST and T wave abnormality, and prolonged QTc

On arrival, an electrocardiogram was obtained (Figure 1), which revealed an undetermined rhythm, nonspecific ST and T wave abnormalities, and prolonged QTc (707 ms) per Bazett formula. Initial labs were remarkable for severe hypokalemia, hypomagnesemia, hypochloremia, elevated creatinine, elevated lactate, and elevated high-sensitivity troponin. Arterial blood gas analysis revealed metabolic alkalosis (Table 1). Radiographic studies were negative for an embolism or intra-abdominal processes.

**Table 1:** Notable values from basic metabolic panel and arterial blood gas on admission

Variable	Patient values	Reference range
Potassium	1.3 mmol/L	3.5-5.0 mmol/L
Magnesium	1.5 mg/dL	1.8-2.3 mg/dL
Chloride	90 mmol/L	98-111 mmol/L
Creatinine	1.17 mg/dL	< 1.03 mg/dL
High sensitivity troponin	992 ng/L	<19 ng/L
Lactate, whole blood	13.6 mmol/L	<2.1 mmol/L
Arterial pH	7.65	7.35-7.45
Arterial bicarbonate	31.9 mmol/L	22-26 mmol/L
Arterial blood partial pressure of carbon dioxide	28.7 mmHg	35-45 mmHg

While in the medical intensive care unit, the patient remained intubated, on targeted temperature management (TTM), and potassium was repleted aggressively. Given the recurrent nature of cardiac arrest and hypokalemia in the context of no known history of tubulopathy, eating disorder, or diuretic use, her presentation was concerning for underlying genetic renal tubular disease. Urine electrolytes were obtained, which revealed spot urine potassium of 16.4 mmol/L, and creatinine of 133 mg/dL (Table 2). We calculated the potassium-to-creatinine ratio, which was suggestive of renal potassium loss. A diuretic screen was performed since it was on our differential and came back negative. At that time, the leading differentials included GS or Bartter syndrome, urine calcium; creatinine levels (Table 2) assisted in the diagnosis of Gitelman's phenotype with urine calcium-to-creatinine ratio of less than 0.7. While admitted, potassium and magnesium levels were closely monitored and repleted aggressively. The patient was also put on potassium-sparing agents, including amiloride and spironolactone. The patient's potassium and magnesium remained stable while on potassium-sparing agents. On day five of her admission, she was extubated and transferred to the general practice unit. The patient underwent successful placement of a dual chamber implantable cardioverter-defibrillator (ICD) for secondary prevention of cardiac arrest. On day ten of admission, she was discharged home with amiloride 5 mg twice daily, magnesium oxide 200 mg daily, spironolactone 25 mg daily, and a follow-up with nephrology. 

**Table 2:** Pertinent values of urine chemistry

Urine electrolytes	Patient values	Reference range
Spot urine potassium	16.4 mmol/L	2-200 mmol/L
Spot urine creatinine	133 mg/dL	1 -300 mg/dL
Spot urine calcium	18.6 mg/dL	0.4-40 mg/dL

## Discussion

This report presents a diagnosis of GS following sudden cardiac arrest in an otherwise asymptomatic individual. GS is a relatively rare genetic disease characterized by renal potassium and magnesium wasting with concomitant metabolic alkalosis and hypocalciuria. It is inherited in an autosomal recessive pattern where the gene encoding the thiazide-sensitive sodium chloride transporter (SLC12A3) is defective, leading to renal salt wasting [1,2]. Sudden cardiac arrest is a relatively rare manifestation of the condition [4,5]. Cardiac arrhythmia results from QT prolongation secondary to profound electrolyte derangements, mainly hypokalemia and hypomagnesemia [6]. Some authors [7] contend that GS is no longer a benign condition as it can lead to dangerous ventricular arrhythmias [3], which can lead to life-threatening sudden cardiac arrest [2], as exemplified in this case presentation. 

Our case demonstrates the importance of establishing an accurate diagnosis in the likely prevention of mortality. As mentioned, the patient’s underlying renal disorder was not appreciated during a similar presentation six months ago with sudden cardiac arrest, severe hypokalemia, hypomagnesemia, and QT prolongation. At the time, she was not diagnosed with GS, and the etiology of prolonged QT was deemed to be multifactorial, including electrolyte derangements and medication-related, as she was on methadone. She was discharged with potassium supplementation, which she did not tolerate because of pill size and stomach discomfort leading to severe hypokalemia and a second cardiac arrest. Had the patient been diagnosed with GS, she could have had appropriate treatment, closer follow-up, and undergone cardiac stratification before discharge, potentially preventing a second out-of-hospital cardiac arrest.

Diagnosing GS can be very challenging due to its high clinical symptom variability. Some patients can be asymptomatic, and others may have milder symptoms, including fever, abdominal pain, vomiting, muscle weakness, tetany, paresthesias, and joint symptoms [2]. Nevertheless, in the appropriate clinical context, characteristic laboratory findings of hypokalemia, hypomagnesemia, metabolic alkalosis, and hypocalciuria can be helpful diagnostic clues raising suspicion for renal tubulopathies. In this report, an accurate diagnosis of electrolyte and acid-base abnormalities led to the initiation of appropriate maintenance therapy and ICD implantation in a woman having suffered a second cardiac arrest. 

Bartter syndrome is another distinct type of renal tubulopathy with a similar presentation s GS, which should be on the differential but can be distinguished from GS with the absence of hypocalciuria [8]. 

GS management usually involves close monitoring, life-long supplementation of potassium and magnesium, and cardiac risk stratification to prevent fatal arrhythmias [2]. This case report also aims to challenge current guidelines for ICD placement as current guidelines do not strongly recommend implantation in reversible arrhythmia causes, such as profound electrolyte derangements. However, there are cases in which it was beneficial in the setting of GS [9]. Similarly, this report demonstrates that ICD could be beneficial for the secondary prevention of fatal arrhythmia in patients with GS.

## Conclusions

This case highlights the importance of including GS in the differential diagnosis of a patient presenting with severe hypokalemia, hypomagnesemia, QT prolongation, and cardiac arrhythmia. As GS can manifest as a sudden cardiac arrest, establi­shing an accurate diagnosis can lead to life-saving interventions such as ICD placement for secondary prevention of cardiac arrhythmias.
